# Combination of RAD001 (everolimus) and docetaxel reduces prostate and breast cancer cell VEGF production and tumour vascularisation independently of sphingosine-kinase-1

**DOI:** 10.1038/s41598-017-03728-3

**Published:** 2017-06-14

**Authors:** Heba Alshaker, Qi Wang, Torsten Böhler, Robert Mills, Mathias Winkler, Tawfiq Arafat, Yoshiaki Kawano, Dmitri Pchejetski

**Affiliations:** 1 School of Medicine, University of East Anglia, Norwich, UK; 2 Faculty of Pharmacy and Medical Sciences, University of Petra, Amman, Jordan; 3 Department of Surgery and Cancer, Imperial College London, London, UK; 4 Norfolk & Norwich University Hospital NHS Foundation Trust, Norwich, UK; 5 Department of Urology, University of Kumamoto, Kumamoto, Japan

## Abstract

Resistance to docetaxel is a key problem in current prostate and breast cancer management. We have recently discovered a new molecular mechanism of prostate cancer docetaxel chemoresistance mediated by the mammalian target of rapamycin (mTOR)/sphingosine-kinase-1 (SK1) pathway. Here we investigated the influence of this pathway on vascular endothelial growth factor (VEGF) production and tumour vascularisation in hormone resistant prostate and breast cancer models. Immunofluorescent staining of tumour sections from human oestrogen receptor (ER)-negative breast cancer patients showed a strong correlation between phosphorylated P70S6 kinase (mTOR downstream target), VEGF and SK1 protein expression. In hormone-insensitive prostate (PC3) and breast (MDA-MB-231 and BT-549) cancer cell lines the mTOR inhibitor RAD001 (everolimus) has significantly inhibited SK1 and VEGF expression, while low dose (5 nM) docetaxel had no significant effect. In these cell lines, SK1 overexpression slightly increased the basal levels of VEGF, but did not block the inhibitory effect of RAD001 on VEGF. In a human prostate xenograft model established in nude mice, RAD001 alone or in combination with docetaxel has suppressed tumour growth, VEGF expression and decreased tumour vasculature. Overall, our data demonstrate a new mechanism of an independent regulation of SK1 and VEGF by mTOR in hormone-insensitive prostate and breast cancers.

## Introduction

Docetaxel is an antineoplastic taxane that is widely used for the treatment and management of patients with breast^1^ and prostate cancers^2^. Docetaxel acts by disrupting microtubule disassembly, resulting in inhibition of mitosis, and ultimately leading to apoptosis^2^. However, drug-related cumulative toxicity, unresponsiveness to docetaxel therapy, and the development of resistance limit its clinical benefits^3–5^. Many tumour cells express vascular endothelial growth factor (VEGF) as a principal regulator of angiogenesis. VEGF induces proliferation of endothelial cells, primarily via the VEGF receptor 2, which in turn increases neovascularisation^6^. Combinations of docetaxel with antiangiogenic compounds such as bevacizumab (anti-VEGF recombinant humanised monoclonal antibody), provide clinical benefit in the treatment of metastatic breast and prostate cancers^7^.

Mammalian target of rapamycin (mTOR) is a protein kinase that is present in two distinct complexes: mTOR complex 1 (mTORC1) and mTOR complex 2 (mTORC2)^8^. The mTORC1 plays a central role in regulating critical cellular processes and deregulation of the mTORC1 signalling pathway is closely associated with tumourigenesis^8, 9^. In breast tumours, mTORC1 also controls angiogenic pathways via VEGF^10^, therefore, mTOR represents a validated target for the treatment of cancer. There is increasing clinical evidence showing promising activity of mTOR inhibitors (rapalogues) against solid tumors and hematologic malignancies^9^. One such rapalogue, RAD001 (everolimus), is an inhibitor of mTORC1 with limited or no effect on mTORC2 activity^11^. RAD001 forms a complex with the intracellular receptor FK506 binding protein 12, which interferes with mTOR activity preventing downstream signalling^9^. Cellular effects of RAD001 include enhancement of apoptosis in some tumour cell lines as well as inhibition of cell proliferation, migration, and angiogenesis in some human cancers^11^. RAD001 is approved for treatment of metastatic renal-cell carcinoma^12^, pancreatic neuroendocrine tumours, and some subtypes of breast cancer^11^.

Increased expression of lipid kinase, sphingosine-kinase-1 (SK1), contributes to cancer progression and oncogenic transformation^13^. Sustained SK1 expression was linked to prostate cancer chemoresistance to low dose taxanes^14^. Breast tumours from patients with locally advanced or metastatic cancers with docetaxel-based chemotherapy had significantly higher SK1 mRNA levels in patients who were non-responders to treatment compared to complete or partial responders^15^. In a previous study, we revealed that RAD001 sensitizes PC3 and DU145 prostate cancer cells and tumours to docetaxel therapy through downregulation of mTOR/SK1 pathway^16^. In breast cancer cells, mTOR inhibition by temsirolimus inhibited angiogenesis via transcriptional inhibition of VEGF production^10^. Here we have investigated the effects of docetaxel/RAD001 combination therapy on VEGF regulation in hormone insensitive prostate and breast cancer cells. Our data from primary human breast tumours and human prostate cancer animal models have shown a correlated expression of SK1 and VEGF, which suggests a common regulatory mechanism. Indeed, in PC3, DU145, MDA-MB-231 and BT-549 cancer cells RAD001 downregulated both SK1 and VEGF through mTOR inhibition. However, contrary to chemoresistance that was shown in PC3 and DU145 cells^16^, SK1 overexpression did not abrogate RAD001-induced reduction in VEGF in prostate (PC3) and breast (MDA-MB-231) cancer models, suggesting that the two pathways are regulated independently. *In vivo*, RAD001-induced VEGF downregulation has significantly decreased prostate tumour vascularisation and acted in synergy with docetaxel to reduce prostate tumour growth. Overall, our results provide novel insights into the mechanisms of synergistic RAD001/docetaxel combination and highlight the potential for the use of this combined therapy in hormone-insensitive prostate and breast cancers.

## Results

### In breast cancer patients VEGF and SK1 protein expression correlate to phosphorylated (p)-P70S6 Kinase (P70S6K) levels

**Figure 1:**
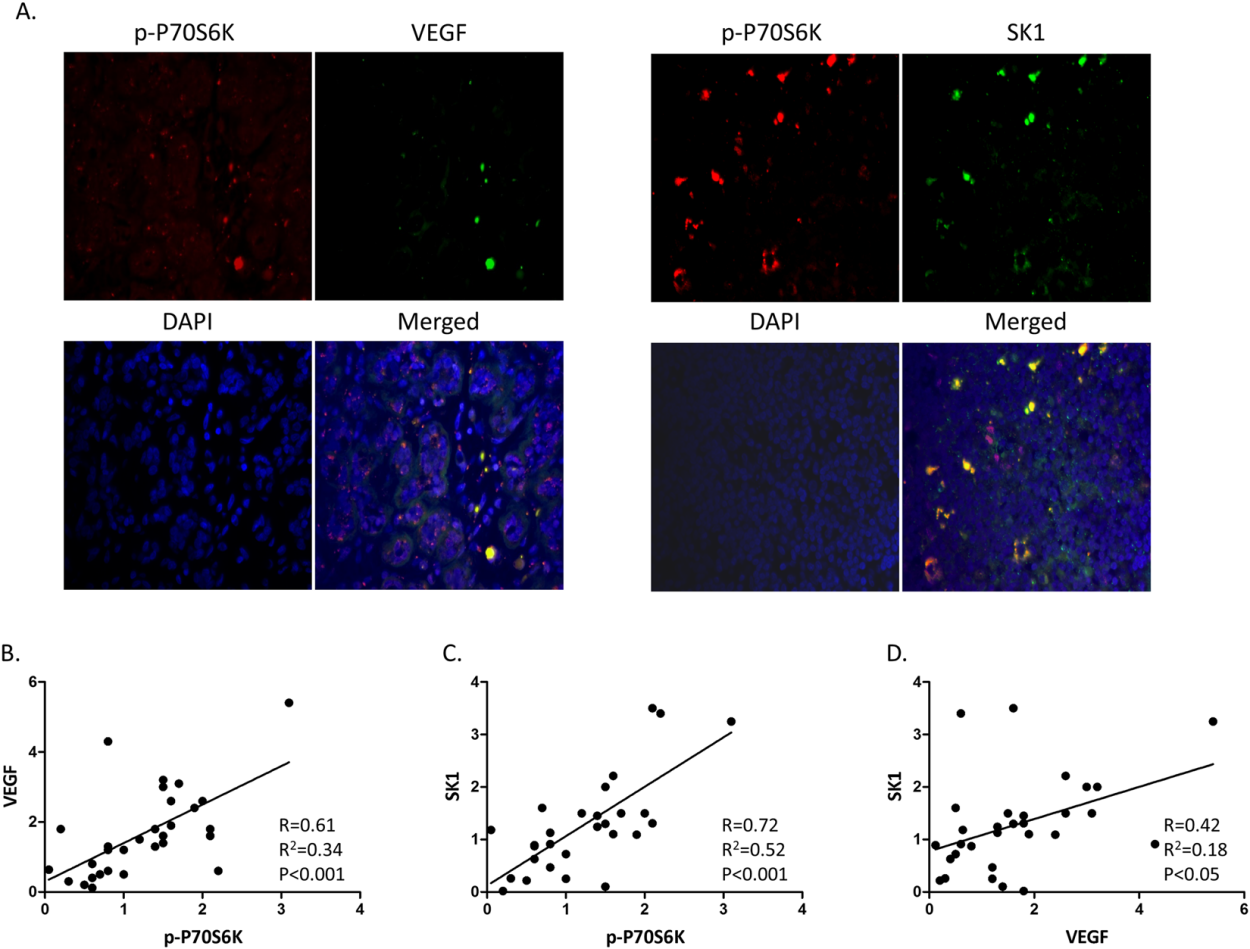
In oestrogen receptor (ER)-negative human breast tumours P70S6K phosphorylation correlates with SK1 and VEGF expression. Human breast tumours (n = 30) were stained with anti-SK1, anti-VEGF and anti-p-P70S6K antibodies. Tumour sections were immunostained as described in materials and methods and fluorescence was visualised using Zeiss Axioplan microscope and quantified using ImageJ software. (**A**) Representative immunofluorescence images of two different tumour sections. Correlation between VEGF and p-P70S6K (**B**) SK1 and p-P70S6K (**C**) and SK1 and VEGF (**D**) in FFPE human ER-negative breast tumours.

To delineate the correlation of the investigated pathways in human patients we used immunofluorescence to quantify relative P70S6K phosphorylation, SK1 and VEGF expression in paraffinised sections of human oestrogen receptor (ER)-negative breast tumours. There was a strong positive correlation between p-P70S6K and VEGF, p-P70S6K and SK1, but less so between SK1 and VEGF (p < 0.001, R = 0.61), (p < 0.001, R = 0.72) and (p < 0.05, R = 0.42), respectively (Fig. 1).

### RAD001 but not docetaxel decreases SK1 and VEGF expression in MDA-MB-231 and PC3 cells

**Figure 2:**
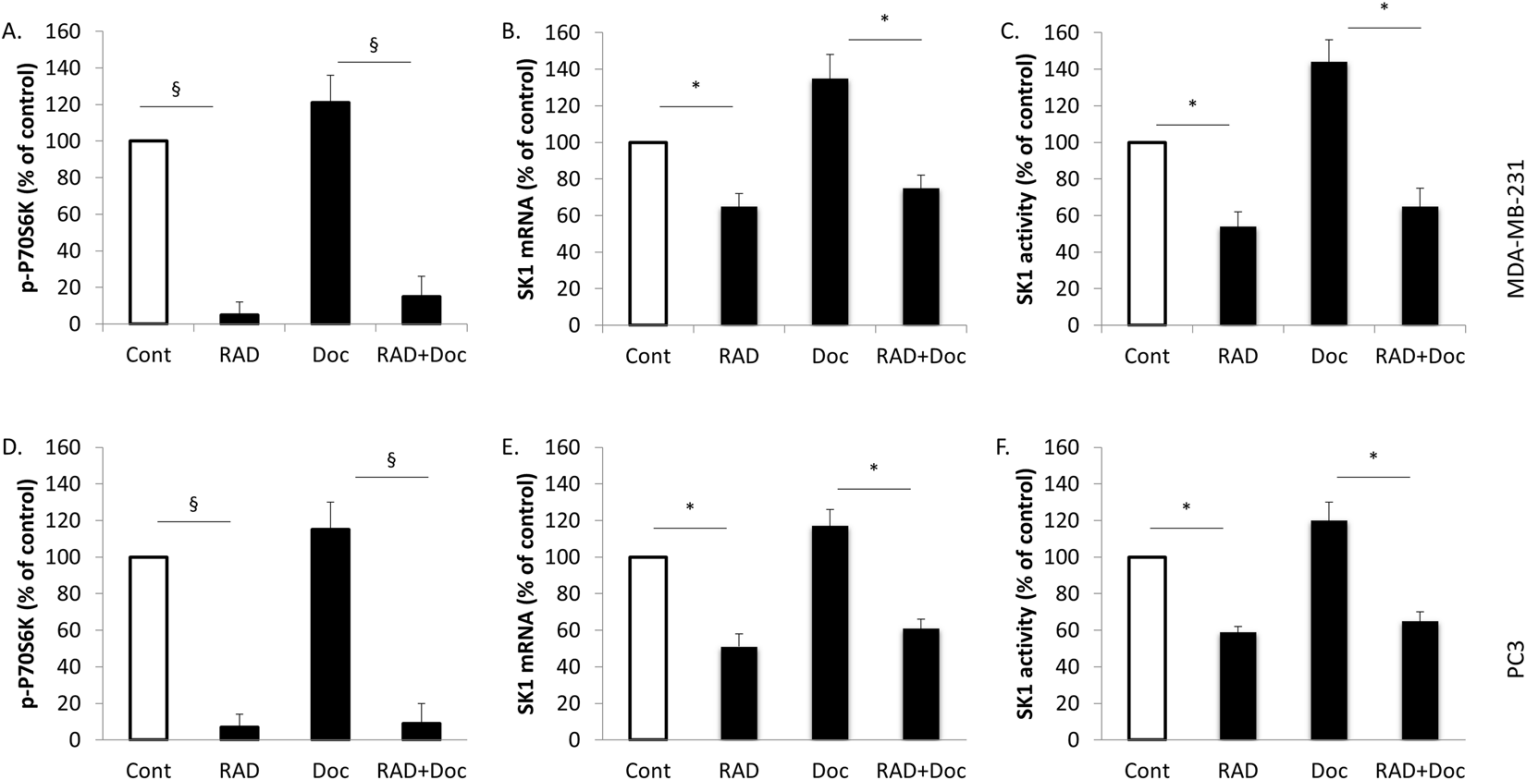
RAD001 but not docetaxel decreases SK1 expression in MDA-MB-231 and PC3 cells. MDA-MB-231 and PC3 cells were starved overnight then incubated with 0.1% DMSO (Cont), 100 nM RAD001 (RAD), 5 nM docetaxel (Doc) and the combination of these drugs (RAD + Doc) for 24 h. (**A**,**D**) P70S6K phosphorylation was measured using ELISA. (**B**,**E**) SK1 expression was determined by qRT-PCR. (**C**,**F**) SK1 activity was measured using radiolabelling. Columns, mean of three independent experiments performed in triplicate; bars, SEM. (^*^P < 0.05; ^**^P < 0.01; ^§^P < 0.001; ns, not significant, P > 0.05).

To elucidate the mechanisms implicated in the effects of RAD001 in combination with docetaxel, we first analysed the effect of docetaxel on the expression of SK1 in MDA-MB-231. Similar to the previous findings in PC3 and DU145 prostate cancer cells^16^, docetaxel did not decrease the levels of P70S6K phosphorylation (a marker for mTOR activity), SK1 mRNA or activity, but rather insignificantly increased them (Fig. 2). In contrast, RAD001 alone or in combination with docetaxel has significantly decreased P70S6K phosphorylation, SK1 mRNA and activity in ER-negative MDA-MB-231 and BT-549 breast cancer cells (Figs 2, S1) and hormone refractory prostate cancer cells PC3 (Fig. 2). We have previously shown a similar effect of RAD001 on SK1 activity and expression in another hormone refractory prostate cancer cell line DU145^16^. In MDA-MB-231 breast cancer cells p-P70S6K ELISA findings were confirmed using Western blotting (Figure S2). In PC3 and DU145 prostate cancer cells, we have previously shown that Western blotting and ELISA show similar results^16^.

**Figure 3:**
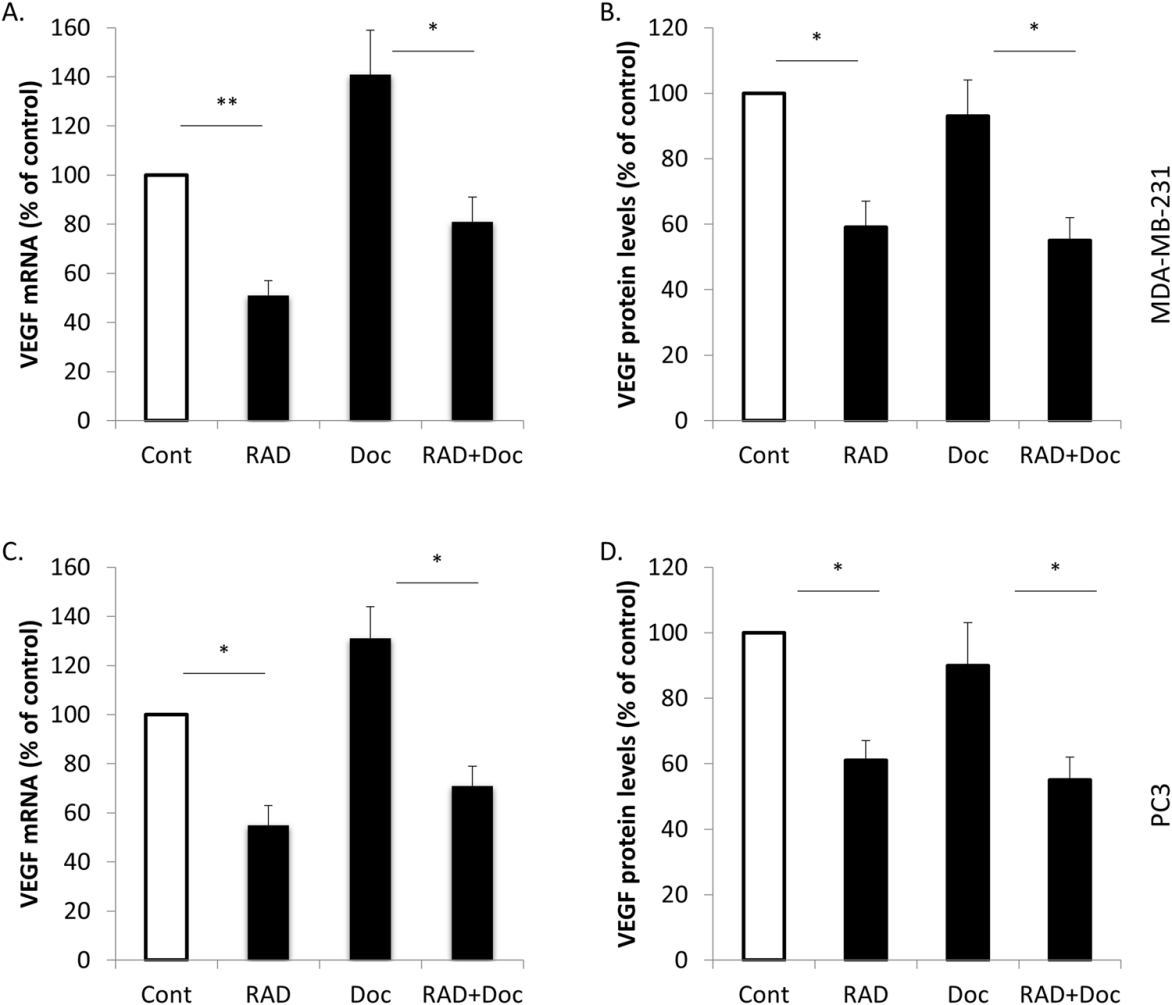
RAD001 but not docetaxel decreases VEGF expression in MDA-MB-231 and PC3 cells. MDA-MB-231 and PC3 cells were starved overnight then incubated with 0.1% DMSO (Cont), 100 nM RAD001 (RAD), 5 nM docetaxel (Doc) and the combination of these drugs (RAD + Doc) for 24 h. (**A**,**C**) VEGF expression was determined by qRT-PCR (**B**,**D**) VEGF protein levels were measured using ELISA. Columns, mean of three independent experiments performed in triplicate; bars, SEM. (^*^P < 0.05; ^**^P < 0.01; ^§^P < 0.001; ns, not significant, P > 0.05).

mTOR regulation of VEGF was suggested in human epidermal growth factor receptor 2 (HER2) positive breast cancer in response to activation of ERB2/PI3K/Akt pathway^17^. Similarly, in prostate cancer, mTOR inhibition with rapamycin was shown to inhibit VEGF secretion^18^, however the combined effects of RAD001 and docetaxel on prostate cancer VEGF production were never studied. Here we show for the first time that in MDA-MB-231, BT-549 and PC3 cells, 5 nM docetaxel increased VEGF mRNA, but did not change secreted VEGF levels (Figs 3, S3). RAD001 alone or in combination with docetaxel significantly decreased both VEGF mRNA levels and VEGF secretion (Figs 3, S3).

### SK1 overexpression increases basal P70S6K phosphorylation and VEGF expression

We have recently shown that in PC3 and DU145 prostate cancer cells mTOR-mediated SK1 expression plays a key role in cancer cell chemoresistance^16^. Previously SK1/sphingosine-1-phosphate (S1P) signalling was implicated in breast cancer angiogenesis and lymphangiogenesis^19^, but the direct link with VEGF was never demonstrated.

**Figure 4:**
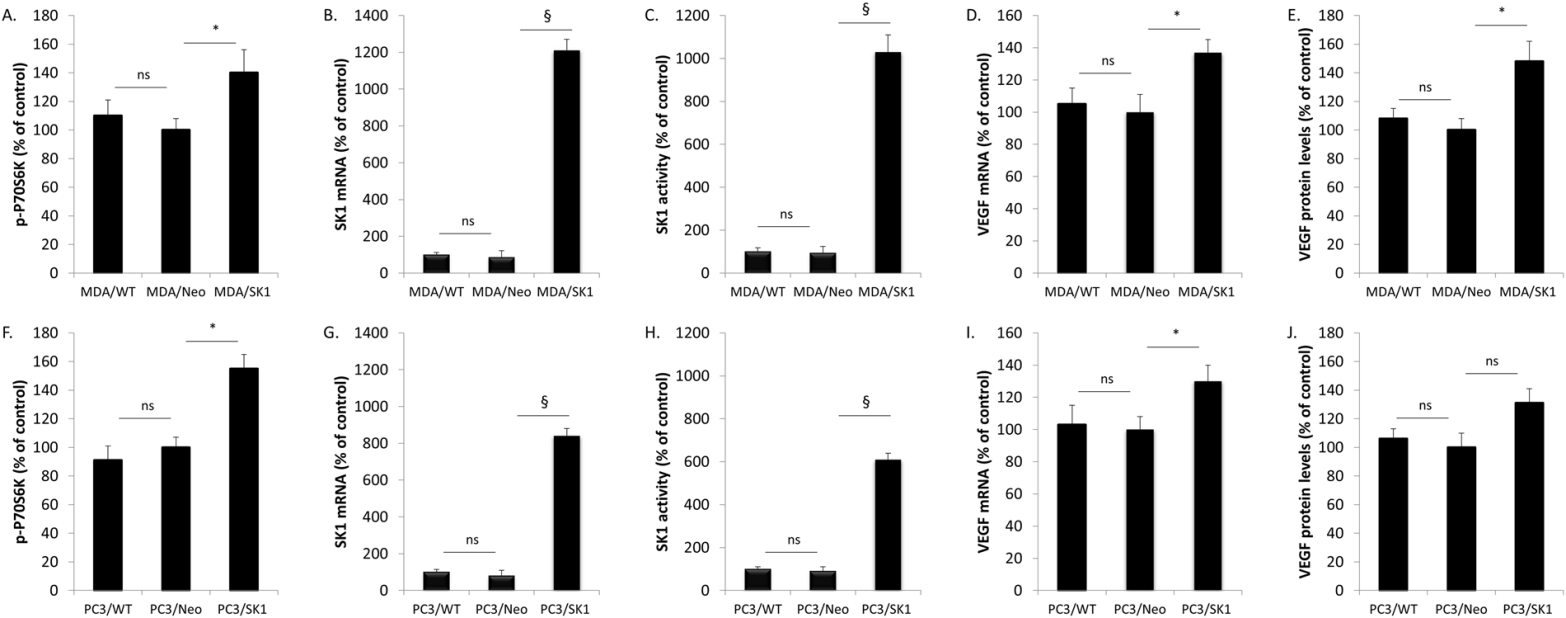
Overexpression of SK1 increases basal P70S6K phosphorylation and VEGF expression. Cell extracts for wild type MDA-MB-231 (MDA/WT) and PC3 (PC3/WT), empty vector-transfected MDA-MB-231 (MDA/Neo) and PC3 cells (PC3/Neo) and MDA-MB-231 and PC3 cells stably transfected with human SK1 (MDA/SK1 and PC3/SK1, respectively) were collected. (**A,F**) P70S6K phosphorylation was measured using ELISA. (**B,G**) SK1 expression and VEGF expression (**D,I**) was determined by qRT-PCR. (**C,H**) SK1 activity was measured using radiolabelling. (**E,J**) VEGF protein levels were measured using ELISA. Columns, mean of three independent experiments performed in triplicate; bars, SEM. (^*^P < 0.05; ^**^P < 0.01; ^§^P < 0.001; ns, not significant, P > 0.05).

To study the effect of SK1 on VEGF production we used MDA-MB-231 cells overexpressing SK1 (MDA/SK1) that had 14- and 11-fold increase in SK1 expression and activity, respectively, in comparison to empty vector-transfected cells (MDA/Neo) (Fig. 4). SK1 overexpression has led to a 40% increase in P70S6K phosphorylation, 37% increase in VEGF expression and 48% increase in VEGF secretion (Figs 4, S4). Similar findings were observed in PC3 prostate cancer cells overexpressing SK1 (Fig. 4). These cells had a 55%, 30% and 31% increase in P70S6K phosphorylation, VEGF expression and VEGF secretion, respectively, in comparison to their empty vector transfected counterparts (Fig. 4). Of note, empty vector transfection did not alter SK1 activity, expression, P70S6K phosphorylation, VEGF expression and secretion in comparison to wild-type cells.

### SK1 overexpression does not affect RAD001-induced VEGF downregulation

**Figure 5:**
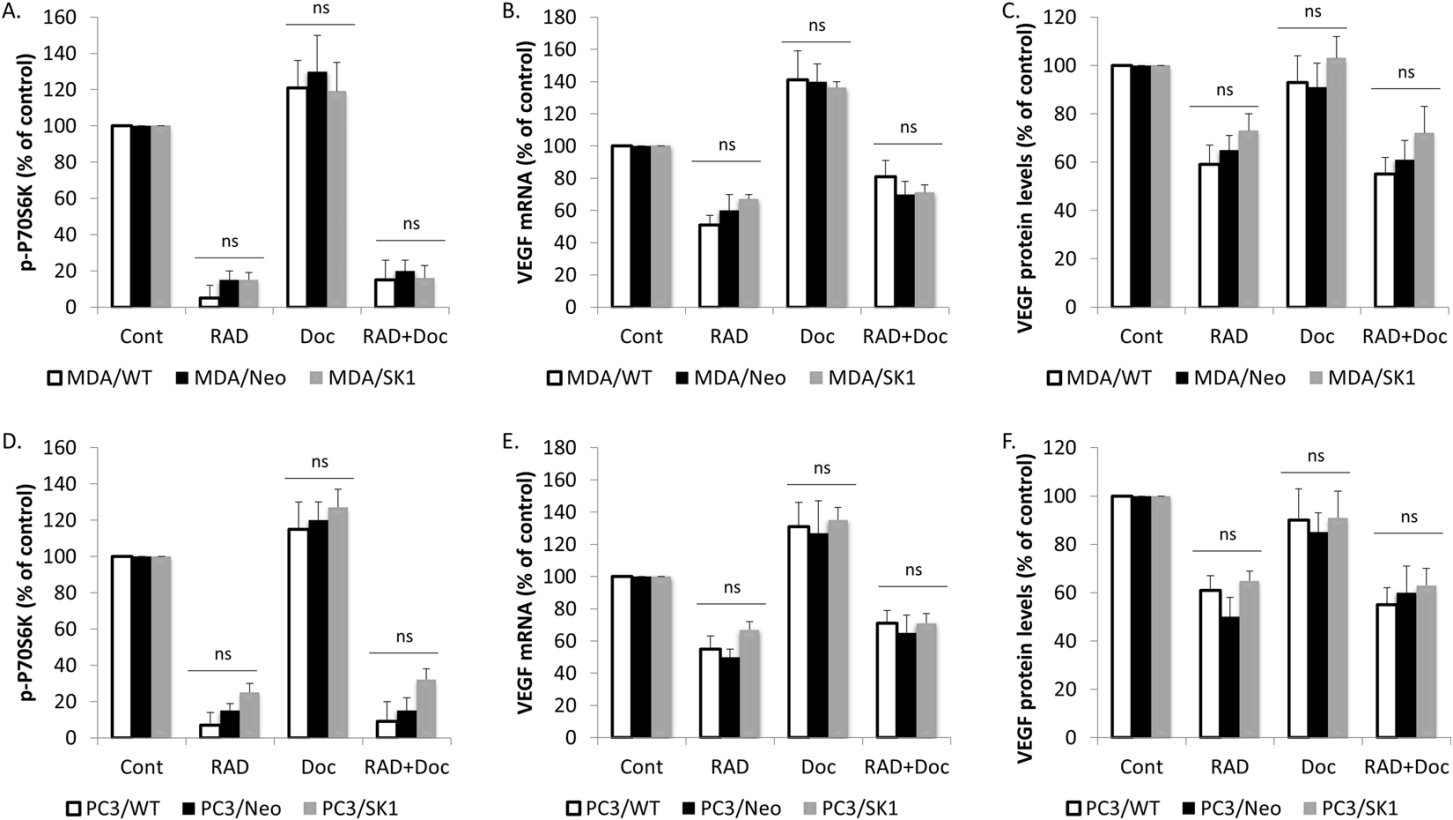
SK1 overexpression does not affect RAD001-induced VEGF downregulation. Wild type, empty vector- and SK1-overexpressing MDA-MB-231 and PC3 cells were starved overnight then incubated with 0.1% DMSO (Control), 100 nM RAD001 (RAD), 5 nM docetaxel (Doc) and the combination of these drugs (RAD + Doc) for 24 h. (**A**,**D**) P70S6K phosphorylation was measured using ELISA. (**B**,**E**) VEGF expression was determined by qRT-PCR. (**C**,**F**) VEGF protein levels were measured using ELISA. Columns, mean of three independent experiments performed in triplicate expressed as relative to control; bars, SEM. (^*^P < 0.05; ^**^P < 0.01; ^§^P < 0.001; ns, not significant, P > 0.05).

We have next investigated the effect of RAD001/docetaxel combination on empty vector- and SK1-overexpressing PC3 and MDA-MB-231 cancer cells. MDA/SK1 cells treated with RAD001 alone or in combination with docetaxel showed similar relative reduction in p-P70S6K levels to MDA/Neo cells, which correlated to same levels of relative reduction in VEGF levels (Figs 5, S4). Similar findings were observed in PC3/SK1 and PC3/Neo cells (Fig. 5).

### Combination of RAD001 and docetaxel blocks prostate tumour growth and decreases tumour vasculature

**Figure 6:**
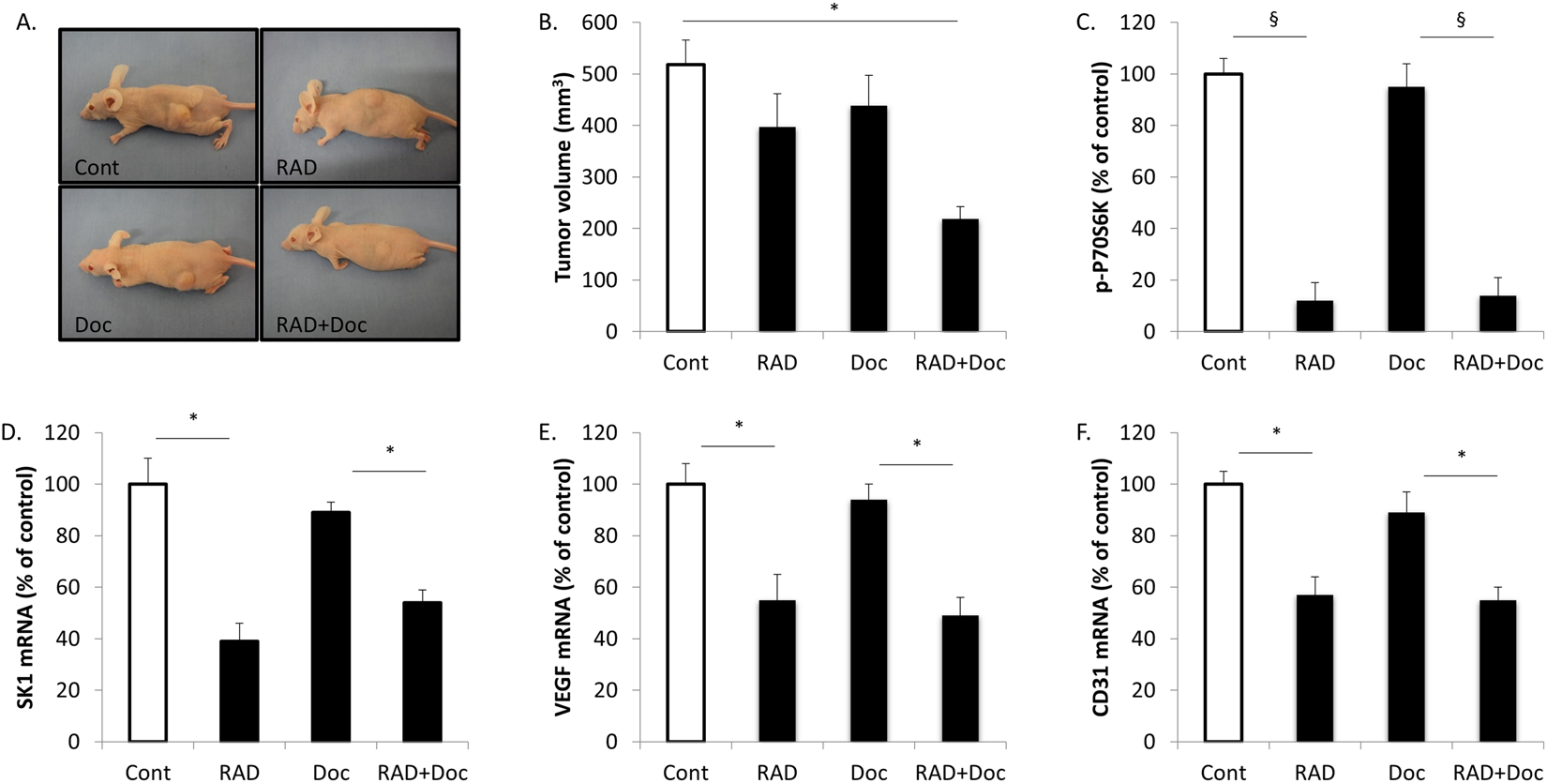
RAD001 decreases tumour vasculature in mouse model of human prostate cancer. Subcutaneous PC3 tumours were established in nude mice as described in materials and methods and allowed to grow for two weeks. Mice were treated with sham (Cont), 5 mg/kg RAD001 (RAD), 5 mg/kg docetaxel (Doc) and combination of these drugs (RAD + Doc) for three weeks. (**A**) Representative images of subcutaneous human tumours in mice after corresponding treatments. (**B**) Tumour volumes at week five. (**C**) P70S6K phosphorylation in tumours was measured using ELISA. (**D–F**) Human SK1 (**D**), VEGF (**E**) and mouse CD31 (**F**) expression was determined by qRT-PCR. Columns, mean values of n = 8; bars, SEM. (^*^P < 0.05; ^**^P < 0.01; ^§^P < 0.001; ns, not significant, P > 0.05).

BALB/c nude mice were subcutaneously implanted with 10^6^ PC3 cells and tumours were left to grow for two weeks. Mice were randomized into groups and treated twice a week with sham intraperitoneal injections, 5 mg/kg docetaxel, 5 mg/kg RAD001 or combination of these drugs for three weeks. As shown in Fig. 6A, treatment with RAD001 has significantly sensitised prostate tumours to small and otherwise inefficient doses of docetaxel achieving a staggering 58% tumour volume reduction in comparison to 23% and 15% by individual RAD001 and docetaxel therapies, respectively (Figs 6A,B, S5). ELISA and qRT-PCR analysis demonstrated that both RAD001 and combined therapy have significantly downregulated tumour p-P70S6K (Fig. 6C), SK1, VEGF and CD31 expression (Fig. 6D–F).

## Discussion

In our recent publication, we have identified a new mechanism of RAD001-induced sensitisation to docetaxel therapy in prostate cancer^16^. We have found that mTOR controls the expression of a proto-oncogene SK1, which in turn mediates chemoresistance. We have shown that combining RAD001 with low dose docetaxel suppresses SK1 activity and acts in synergy in human prostate cancer mouse model drastically reducing tumour size^16^.

The mTOR signalling has high clinical relevance in human prostate and breast cancers. In prostate cancer patients, there was a statistically significant decrease of mTOR and PS6K expression after hormonal deprivation therapy^20^. High expression of nuclear phosphorylated mTOR in ER-negative breast carcinomas suggests that mTOR inhibitors may be effective in at least some patients with this subtype of breast cancer^21^. Previously we reported that SK1 expression was significantly higher in lymph nodes derived from ER-negative breast tumours when compared to ER-positive^22, 23^. Our previous findings were corroborated by a recent report^15^ showing that ER-negative breast tumours express higher SK1 mRNA compared to ER-positive tumours, which correlate with poor overall and progression-free survival in breast cancer patients. Additionally, higher SK1 mRNA levels were observed in breast cancer patients who were non-responders to docetaxel treatment compared to complete or partial responders^15^. Immunofluorescent analysis of ER-negative breast tumours shows a strong positive correlation between p-P70S6K/VEGF and p-P70S6K/SK1 and albeit smaller correlation between SK1 and VEGF (Fig. 1).

Here we show that similar to hormone refractory prostate cancer cells PC3 and DU145^16^, in ER-negative breast cancer cells MDA-MB-231 and BT-549 RAD001 alone or in combination with docetaxel, but not docetaxel alone, can downregulate p-P70S6K and SK1 signalling (Figs 2, S1), suggesting the presence of a similar pathway in breast cancer. A wealth of evidence suggests an important role for the mTOR/VEGF pathway in HER2 positive breast cancer after the initial finding that rapamycin can dramatically inhibit breast tumour growth and vascularisation^17^. In the HER2 gene-amplified breast cancer cells, temsirolimus inhibited VEGF production *in vitro* under both normoxic and hypoxic conditions^10^. Authors observed a similar effect in MDA-MB-231 cells^10^. Similarly, in PC3 prostate cancer cells RAD001 reduced the secretion of VEGF^24^. Interestingly, in one publication authors have shown that docetaxel increases VEGF expression in breast and prostate cancer cells^7^. This provides rationale for the use of anti-VEGF therapy together with docetaxel in these cancers. In this context, RAD001/docetaxel combination was previously tested in ER-negative breast cancer stem cells^25^, but its effect on VEGF-mediated tumour vascularisation was not elucidated. Here we show that in both cell lines, docetaxel has increased VEGF mRNA levels, but did not change the levels of secreted protein, while RAD001 alone or in combination with docetaxel significantly decreased these levels (Fig. 3).

We have previously shown that increased SK1 expression is observed in aggressive human prostate tumours^26, 27^ and ER-negative breast cancers^22, 23^. There is a significant link between SK1 and angiogenesis, however, most studies agree that the mechanism is VEGF-independent and is mediated by SK1-produced S1P and its extracellular receptors^13^. One publication has shown that S1P produced by SK1 increases the level of VEGF mRNA in human umbilical vein endothelial cells (HUVECs)^28^, however in breast cancer cells SK1/S1P activation was sufficient to induce angiogenesis without the involvement of VEGF pathway^19^. Our data suggest that while in both PC3 and MDA-MB-231 cancer cells SK1 overexpression slightly increases basal VEGF levels (Fig. 4), it does not affect RAD001-induced VEGF reduction (Fig. 5).

Since the initial hallmark paper demonstrating the ability of rapamycin to downregulate tumour VEGF levels^29^, the ability of mTOR inhibitors to elicit antiangiogenic effects by downregulation of VEGF, made them key candidates for the investigation of new combined chemotherapy regimens. RAD001 was shown to have an additive effect to docetaxel in reducing the volumes of the xenograft tumours induced in mice by MDA-MB-231 stem cells^25^. Similarly, we have recently shown that combined RAD001 and docetaxel treatment had synergy in reducing PC3 tumour growth in nude mice^16^. However, in neither system VEGF expression was studied. Here we evaluated the effect of RAD001 alone or in combination with docetaxel on prostate tumour VEGF production and tumour vascularisation. Treatment with RAD001 has significantly sensitised PC3 prostate tumours to small and otherwise inefficient doses of docetaxel achieving a staggering 58% tumour volume reduction in comparison to 23% and 15% by individual RAD001 and docetaxel therapies, respectively (Figs 6, S5). All therapies containing RAD001 have significantly downregulated tumour p-P70S6K levels, SK1, VEGF and CD31 expression (Fig. 6). In ER-negative breast tumours, p-P70S6K/VEGF and p-P70S6K/SK1 have exhibited high degree of correlation, however, SK1/VEGF had a lower degree of correlation, suggesting the absence of cross-regulation (Fig. 1).

In conclusion, we show that both in prostate and breast cancers VEGF and SK1 are independently regulated by mTORC1. mTOR inhibition significantly reduces VEGF and tumour vascularisation and complements docetaxel therapy. Our data provide rationale for potential use of mTOR inhibitors in combination with docetaxel in these cancers.

## Methods

### Patients’ samples

Archival paraffin-embedded tissue from 30 patients with ER-negative primary breast tumours was obtained from Imperial College NHS Trust tissue bank. Prior to donating their samples to the bank, all patients filled an informed consent for their subsequent use in research projects and publications. The study was approved by National Research Ethics Service and performed in accordance with ethical guidelines. None of the patients received neo-adjuvant therapy. Clinicopathological details of the patients enrolled in this study are listed in Table S1. All samples were formalin-fixed and paraffin-embedded (FFPE). Four sections (5-µm-thick) were macro dissected from the FFPE blocks with trimming of excess paraffin. Only tissue containing at least 70% of tumour was used for immunofluorescence.

### Cell lines and cell culture

Androgen insensitive prostate cancer cell line (PC3) was obtained from DSMZ (Braunschweig, Germany). Breast cancer cell lines MDA-MB-231 and BT-549 were purchased from ATCC (Manassas, VA, USA). PC3/SK1 and MDA/SK1 were derived from parental cell lines through stable transfection with human SK1^16, 30–33^. Cells were maintained in tissue culture flasks or plastic dishes in a humidified atmosphere of 5% CO_2_ at 37 °C using Roswell Park Memorial Institute (RPMI) 1640 for PC3 or Dulbecco’s Modified Eagle’s Medium (DMEM) for MDA-MB-231 and BT-549 supplemented with 10% heat-inactivated fetal bovine serum (FBS) (Sigma‐Aldrich, UK), 50 U/ml penicillin, 50 μg/ml streptomycin and 2 mM glutamine (Sigma‐Aldrich, UK). In the case of stably transfected cancer cell lines, the growth medium was supplemented with 1 mg/ml Geneticin sulfate (G418, Santa Cruz Biotechnology, Heidelberg, Germany). Cell lines were routinely verified by morphology and growth curve analysis and routinely screened for mycoplasma infection (using MP0035 Lookout, Sigma). All experiments were conducted in the absence of serum. Cells were seeded to be 80% confluent by the end of the treatment and were treated as indicated in figures’ legends. Cell lines were kept in culture for up to 30 passages.

### Reagents

Silica gel 60 high-performance TLC plates were from VWR (West Chester, PA, USA), and [γ-^32^P]-ATP was purchased from Perkin-Elmer (Waltham, MA, USA). RAD001 (everolimus) from Selleckchem (Newmarket, UK). Other reagents and chemicals used were purchased from Sigma-Aldrich (Dorset, UK) unless otherwise specified.

### ELISA for p-P70S6K (Thr389)

For the measurements of p-P70S6K, cancer cell or tumour lysates were prepared and ELISA was conducted as previously described^16^. Equal amount of lysates was loaded onto PathScan p-P70S6K plates and PathScan total P70S6K plates (Cell Signaling, Danvers, USA), and the assay was performed according to manufacturer’s instructions. Levels of p-P70S6K were normalized to corresponding total P70S6K levels.

### RNA extraction, cDNA synthesis and qRT-PCR

Isolation of total RNA from prostate cancer cell lines and mouse tumours was performed using the RNeasy Mini kit (Qiagen, Valencia, CA, USA) as per manufacturer’s instructions. RNA quantity and purity was measured using a NanoDrop 2000c Spectrophotometer (Thermo Fisher Scientific, Loughborough, UK). Reverse transcription was performed using Precision nanoScript™ Reverse transcription kit (PrimerDesign Ltd, Southampton, UK). qRT-PCR was done as previously described^22, 23^. Ct values were exported and analysed using qbase software (Biogazelle NV, Zwijnaarde, Belgium).

### Sphingosine kinase 1 assay

SK1 assay was performed using radiolabelling as previously described^23, 34^, in conditions favouring SK1 activity and inhibiting SK2 activity.

### Animal study

Animal study was performed as previously described^16, 31, 33^. Briefly, subcutaneous human prostate cancer xenografts were established in BALB/c nude male mice by subcutaneous injection of 1*10^6^ PC3 cells. Two weeks after implantation, mice were randomized into treatment groups and treated twice a week for three weeks with: i.p. injections of vehicle (control), 5 mg/kg docetaxel, 5 mg/kg RAD001 and a combination of these drugs. One day after the last treatment, all mice were euthanized. Mice primary tumours were frozen and processed for radiolabelling, ELISA and qRT-PCR analysis. Animal studies were performed under the Home Office license and carried out in in accordance with the institutional guidelines and regulations for animal welfare (University of East Anglia) and NC3Rs (Replacement, Reduction and Refinement) guidelines. The experiments were carried out in accordance to the protocols section 19B of the Home Office licence and were approved by the University of East Anglia animal welfare committee.

### Immunofluorescent staining of human tumours

Fluorescent immunostaining was performed using 5-µm-thick FFPE tissues of human ER-negative primary breast tumours. Tissues were deparaffinised in xylene and were rehydrated in graded ethanol (100, 95, 80, 70, 50 and 30% v/v). Antigen retrieval was performed by incubation with 20 µg/ml Proteinase K for 30 min at 37 °C. Sections were incubated in blocking buffer (5% v/v normal donkey serum in PBS) for 2 h at room temperature then probed with antibodies against SK1, VEGF (Abcam, UK) and p-P70S6K (Thr389) (SantaCruz Biotechnology, UK) overnight in a humidified chamber at 4 °C. Slides were washed in 0.1% Tween-20 in PBS (pH 7.2–7.4) and were incubated with secondary Alexa Fluor 488-conjugated anti-rabbit IgG, (Abcam, UK), and Alexa Fluor 647-conjugated anti-goat IgG (ThermoFisher Scientific, UK), for 2 h at room temperature. Cell nuclei were counterstained using DAPI, and fluorescence was visualised using Zeiss Axioplan immuno fluorescent microscope. Image analysis was performed using the ImageJ software (http://rsb.info.nih.gov/ij) and the threshold was set according to the negative control captured image.

### Statistical analysis

Data are presented as the mean values of at least three independent experiments normalised to control ± standard error of the mean (SEM) calculated using GraphPad Prism. Statistical significance between two groups was conducted by unpaired Student’s t test. Comparisons between the means of more than two groups were assessed using one-way ANOVA analysis followed by a Tukey’s test (95% confidence). P value of < 0.05 is considered statistically significant. The Pearson’s correlation coefficient was calculated between the expression levels of two target genes.

## Electronic supplementary material

Supplementary Information
