# Supplementary material for: Combination of RAD001 (everolimus) and docetaxel reduces prostate and breast cancer cell VEGF production and tumour vascularisation independently of sphingosine-kinase-1
Source: Sci Rep. 2017 Jun 14;7:3493. doi: 10.1038/s41598-017-03728-3 (PMC5471177; doi:10.1038/s41598-017-03728-3)
Supplement: Supplementary file 1 — Supplementary Information [file 41598_2017_3728_MOESM1_ESM.pdf]

Combination of RAD001 (everolimus) and docetaxel reduces prostate and breast cancer cell VEGF production and tumour vascularisation independently of sphingosine-kinase-1

Heba Alshaker[1,2*], Qi Wang[1], Torsten Böhler[3], Robert Mills[4], Mathias Winkler[3], Tawfiq Arafat[2], Yoshiaki Kawano[5], Dmitri Pchejetski[1*]

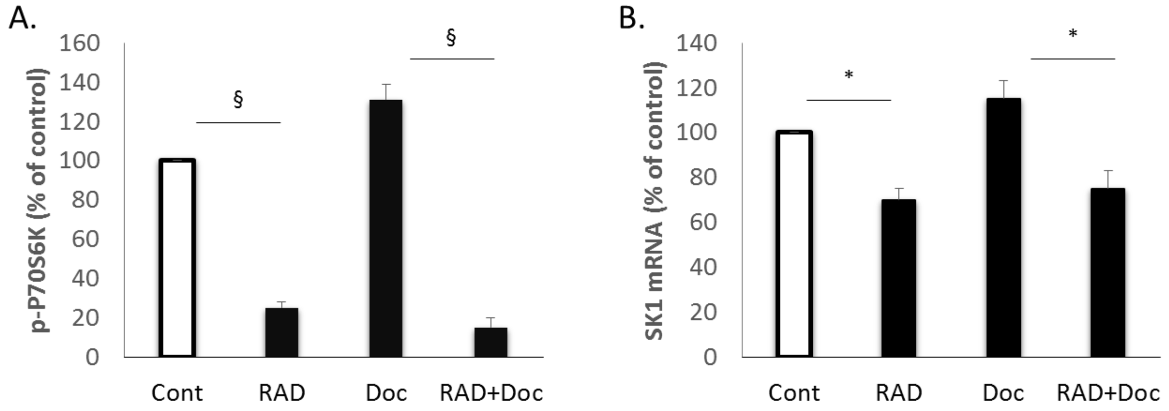

Figure S1. RAD001 but not docetaxel decreases SK1 expression in BT-549 cells. BT-549 cells were starved overnight then incubated with 0.1% DMSO (Cont), 100nM RAD001 (RAD), 5nM docetaxel (Doc) and the combination of these drugs (RAD+Doc) for 24h. **A.** P70S6K phosphorylation was measured using ELISA. **B.** SK1 mRNA expression was determined by qRT-PCR. Columns, mean of three independent experiments performed in triplicate; bars, SEM. (*, P<0.05; §, P<0.001; ns, not significant, P>0.05).

Figure S2. RAD001 decreases P70S6K phosphorylation in MDA-MB-231. MDA-MB-231 cells were starved overnight then incubated with 0.1% DMSO (Cont), 100nM RAD001 (RAD), 5nM docetaxel (Doc) and the combination of these drugs (RAD+Doc) for 24h. Cell extracts were loaded on acrylamide mini gel and probed for phosphorylation of P70S6K and GAPDH.

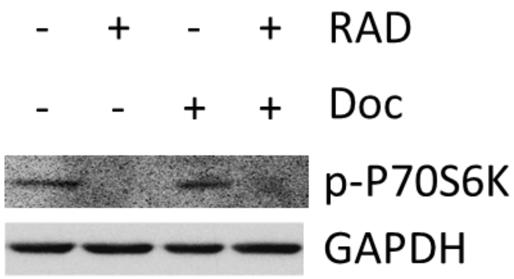

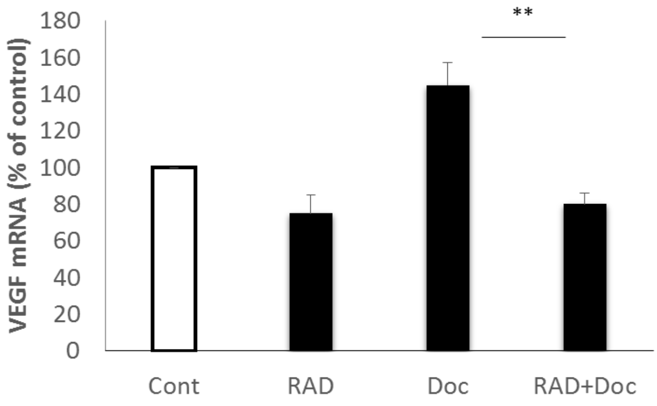

Figure S3. RAD001 but not docetaxel decreases VEGF expression in BT-549 cells. BT-549 cells were starved overnight then incubated with 0.1% DMSO (Cont), 100nM RAD001 (RAD), 5nM docetaxel (Doc) and the combination of these drugs (RAD+Doc) for 24h. VEGF mRNA expression was determined by qRT-PCR. Columns, mean of three independent experiments performed in triplicate; bars, SEM. (*, P<0.05; **, P<0.01; §, P<0.001; ns, not significant, P>0.05).

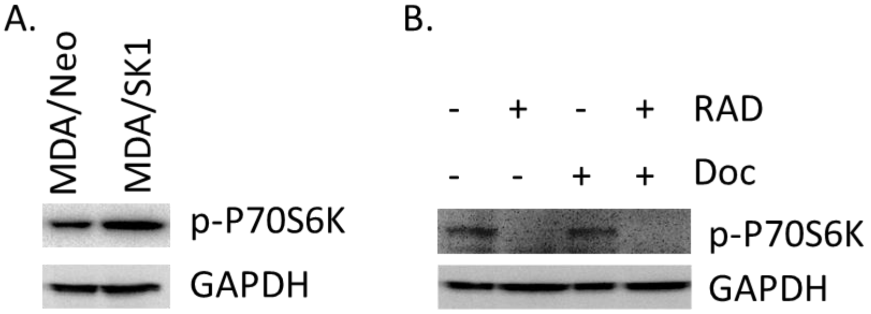

Figure S4. SK1 overexpression does not block RAD001-mediated inhibition of mTOR in MDA-MB-231 cells. A. Cell extracts for empty vector-transfected MDA-MB-231 cells (MDA/Neo) and MDA-MB-231 cells stably transfected with human SK1 (MDA/SK1) were loaded on acrylamide mini gel and probed for p-P70S6K and GAPDH. **B.** MDA/SK1 cells were starved overnight then incubated with 0.1% DMSO (Cont), 100nM RAD001 (RAD), 5nM docetaxel (Doc) and the combination of these drugs (RAD+Doc) for 24h. Cell extracts were loaded on acrylamide mini gel and probed for p-P70S6K and GAPDH.

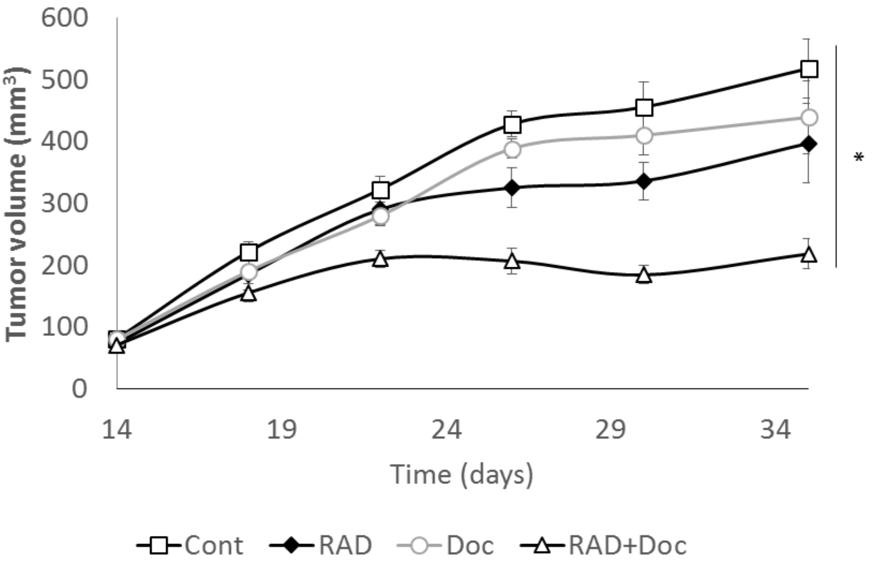

Figure S5. RAD001 decreases tumour growth in mouse model of human prostate cancer. Subcutaneous PC3 tumours were established in nude mice as described in materials and methods and allowed to grow for two weeks. Mice were treated with sham (Cont), 5mg/kg RAD001 (RAD), 5mg/kg docetaxel (Doc) and combination of these drugs (RAD+Doc) for three weeks. Tumour sizes were measured every 4 days and volumes calculated as 4/3*3.14*a*c*c where a is a long diameter and c is a short diameter. Columns, mean values of n=8; bars, SEM. (*, P<0.05; **, P<0.01; §, P<0.001; ns, not significant, P>0.05).

Table S1. Patient characteristics with clinicopathological parameters of oestrogen receptor (ER)-negative breast cancer patients

Characteristics	Patients (n[%])
**Age (Years)**	
30-39	6 [20]
40-49	7 [23]
50-59	8 [27]
≥60	9 [30]
**Nodal status**	
1-3	16 [53]
≥4	14 [47]
**Tumour size (mm)**	
0-20	13 [43]
21-50	12 [40]
≥51	5 [17]
**Tumour grade**	
II	6 [20]
III	24 [80]
**Progestrone receptor status**	
Positive	5 [17]
Negative	25 [83]
**HER2 status**	
Positive	10 [33]
Negative	20 [67]
**Histologic type**	
Invasive ductal carcinoma	30 [100]
